# 3D printing of microbial communities: A new platform for understanding and engineering microbiomes

**DOI:** 10.1111/1751-7915.14168

**Published:** 2022-12-13

**Authors:** Ravinash Krishna Kumar, Kevin R. Foster

**Affiliations:** ^1^ Centre for Bioengineering, School of Engineering and Materials Science Queen Mary University of London London UK; ^2^ Department of Chemistry University of Oxford Oxford UK; ^3^ Department of Biochemistry University of Oxford Oxford UK; ^4^ Department of Biology University of Oxford Oxford UK

## Abstract

3D printing has emerged as a powerful way to produce complex materials on‐demand. These printing technologies are now being applied in microbiology, with many recent examples where microbes and matrices are co‐printed to create bespoke living materials. Here, we propose a new paradigm for microbial printing. In addition to its importance for materials, we argue that printing can be used to understand and engineer microbiome communities, analogous to its use in human tissue engineering. Many microbes naturally live in diverse, spatially structured communities that are challenging to study and manipulate. 3D printing offers an exciting new solution to these challenges, as it can precisely arrange microbes in 3D space, allowing one to build custom microbial communities for a wide range of purposes in research, medicine, and industry.

## Graphical abstract

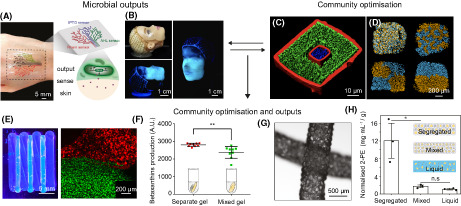

Proposed as early as 1945 in science fiction (Leinster, 1945), it was not until the 1980s that 3D printing technologies such as stereolithography were formally described (Jakus, 2019; Kodama, 1998). Initially used for prototyping in the manufacturing industry, 3D printing is now widely used to make custom parts in industries including aviation, automotive, and consumer goods (Bogers et al., 2016; Ngo et al., 2018). In parallel to its development in manufacturing, it was realised that 3D printing had significant potential in medicine (Gu et al., 2020). The cells within our tissues and organs are arranged precisely in three‐dimensional space, and correct structure is critical to their functioning. This spatial complexity makes it challenging to grow many tissues and organs, and yet there is a great demand to generate tissue and organ replacements for when they fail (Murphy & Atala, 2014). 3D printing offers a potential solution to this problem as, in principle, different cell types can be patterned in the necessary configurations for the normal functioning of a given tissue. This ambitious goal has driven the field of bioprinting, which is the controlled patterning of cells using a printing technology. Since its inception, bioprinting has rapidly developed from two‐(Klebe, 1988) to three‐dimensional patterns by adapting different types of 3D printing technologies (e.g. droplet or extrusion printing) to print cells embedded within biocompatible (not harmful to living tissue) hydrogels (Gu et al., 2020; Zhou et al., 2020). Pioneering experiments have now shown that one can transplant 3D‐printed cellular constructs into animal hosts, where these constructs integrate (into host tissue) and survive for weeks (Kang et al., 2016).

Many challenges remain before 3D‐printed human organs are fit for clinical use. Nevertheless, innovative work has made it clear that one can readily print live cells in three‐dimensional patterns. However, it was only recently that these technologies have been applied to microbes. Thus far, the focus in microbiology has been quite different to that in human cell printing: rather than seeking to recreate microbial communities in their natural states, it was realised that microbes can be combined with abiotic matrices to create novel living materials (Duraj‐Thatte et al., 2021; González et al., 2020; Huang et al., 2019; Johnston et al., 2020; Lehner et al., 2017; Liu et al., 2018; Ou et al., 2022; Schaffner et al., 2017). Bacteria, in particular, can be mixed with biocompatible aqueous solutions that usually contain nutrients and chemical components to form a self‐supporting hydrogel. In this way, bacteria can be patterned into complex 3D architectures, with a wide range of potential uses. For example, Liu et al. developed living sensors from patterning hydrogels and engineered *Escherichia coli* (Liu et al., 2018). Their method allowed the printing of wearable materials, where *E. coli* within the printed hydrogel were genetically modified to sense chemical inducers (*N*‐acyl homoserine lactone, isopropyl β‐D‐1‐thiogalactopyranoside and rhamnose) embedded in human skin by outputting a fluorescence signal in response to the inducers (Figure 1A). An exciting application of this technology could be to produce implantable or digestible sensors, which could monitor disease biomarkers in patients with chronic conditions, such as diabetes or obesity. Another possibility is to use printed bacteria to create wound dressings. Schaffner et al. used the cellulose‐producing bacteria *Acetobacter xylinum* to generate three‐dimensional cellulosic structures (Figure 1B) (Schaffner et al., 2017). Cellulose is a difficult material to purify and spatially manipulate but has desirable physical/mechanical properties for biomedical applications (Seddiqi et al., 2021). Remarkably, the cellulose produced by *A. xylinum* templated the original printed hydrogel structure, raising the possibility of making precise cellulosic structures that can serve as skin grafts.

**FIGURE 1:**
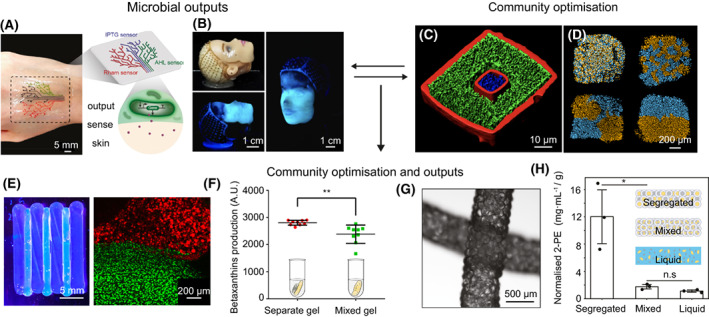
Examples of 3D‐printed microbial systems and their applications. (A) Printed living tattoo containing ‘sensing’ *E. coli* on human skin reproduced from Liu et al. (2018). (B) Printed *A. xylinum* onto a model face producing a cellulosic mask; reproduced from Schaffner et al. (2017). (C) Spatially segregated microcolonies by 3D printing. *S. aureus* (blue) is separated from *P. aeruginosa* (green) by a porous gelatin barrier (red). Panel is reproduced from Connell et al. (2013). (D) Printing communities of two strains of *E. coli* (blue and orange), with different degrees of spatial mixing; reproduced from Krishna Kumar et al. (2021). (E) A printed community where the species *E. coli* and *S. cerevisiae* are segregated (left panel) and a higher resolution fluorescence microscopy image of the printed construct (right panel) with *E. coli* in green and *S. cerevisiae* in red. (F) A plot comparing the production of the food colourant betaxanthin against hydrogels with segregated or mixed communities of *E. coli* and *S. cerevisiae*; ** (*p* = 0.0054) for an unpaired *t* test with Welch's correction. (E, F) Reproduced from Johnston et al. (2020). (G) Printed scaffolds of connected microgels containing *E. coli* and *M. guilliermondii*. (H) A plot of normalised 2‐phenylethanol production by communities that are segregated (heterogenous scaffolds), mixed (homogenous scaffolds), or in liquid culture; * (*p* = 0.021) and n.s. (*p* = 0.069) for an unpaired two‐tailed student's t test. (G, H) Reproduced from Ou et al. (2022).

Other potential applications for printed microbial materials include roles in bioremediation (Schaffner et al., 2017) and chemical production (Duraj‐Thatte et al., 2021; Huang et al., 2019). However, to date, most methods have focused on printing a single strain of bacteria or other microbe. While this can work well in some contexts, the solitary life of a microbe does not reflect the natural state for most microbial communities, where different strains and species live alongside one another (Flemming & Wuertz, 2019). These complex communities are important for health (Fan & Pedersen, 2021), agriculture (Philippot et al., 2013), industry (Wu et al., 2019), and ecosystem services (such as biogeochemical cycling) (Rousk & Bengtson, 2014). A key barrier to understanding such communities, and thus to controlling them, is that they contain many interacting species, whose precise arrangement at micron scales can be critical for how a given community behaves (Wessel et al., 2013). For example, whether different strains of bacteria are well‐mixed or growing in distinct patches can determine whether one strain goes extinct, or both coexist (Krishna Kumar et al., 2021). Moreover, there is considerable variation in the spatial structure observed within different communities. The oral microbiota comprises of many species structured into distinct clonal patches (Welch et al., 2016; Wilbert et al., 2020), whereas the gut microbiota appears more well‐mixed (Welch et al., 2017; Whitaker et al., 2017). There is a need, therefore, for technologies that both recreate and manipulate the natural structures of microbial communities. This leads us to our proposed paradigm: 3D printing can generate diverse, patterned microbial communities to better understand and harness microbes for a wide range of applications.

There is much to explore. Most fundamentally, one can arrange microbial species in different patterns to see how this affects their growth and survival. Pioneering work from Connell et al. did just this, using multiphoton lithography to arrange cells of two bacterial species at micrometre length‐scales in gelatin (Figure 1C) (Connell et al., 2013). This work revealed that adding a picolitre shell of *Pseudomonas aeruginosa* around a microcolony of *Staphylococcus aureus* was enough to protect the *S. aureus* microcolony from antibiotics diffusing into the printed community. This protection arose because *P. aeruginosa* makes an enzyme that breaks down the antibiotics, thereby allowing *S. aureus* to persist.

In our own work, we have asked how changing the patterns of bacterial strains influences community properties. To do this, we developed a 3D droplet‐printing method to arrange different bacterial strains at micrometre length scales and follow them as they grow and interact (Krishna Kumar et al., 2021) (Figure 1D). With this, we printed different strains of *E. coli* that compete by using protein toxins that target one another. This work revealed that the spatial arrangement of the strains at the micron scale can be critical for ecological outcomes, including which bacterial genotypes persisted, how strains could shield each other from toxins and how productive a community was. Importantly, the outcomes of these experiments differed to work performed at much larger scales on agar plates and liquid cultures (Mavridou et al., 2018), including which strain won the competition. This finding highlighted the importance of studying microbial communities at their natural scales.

Fine‐scale spatial structuring has also proven valuable in industrial contexts. Johnston et al. used 3D printing to build and optimise a two‐species community capable of synergistically producing the food colourant betaxanthin (Figure 1E) (Johnston et al., 2020). In this community, *E. coli* converts glucose to a dopamine precursor (L‐DOPA), which is then converted by *Saccharomyces cerevisiae* into betaxanthin. By printing the two species in different spatial arrangements, the authors found that they could increase betaxanthin production by spatially segregating *E. coli* and *S. cerevisiae* (Figure 1F). They hypothesised that, when segregated, the species in question are not in direct competition for nutrients and so are better able to coexist and sustain production of betaxanthin. Another example uses this idea of spatial segregation to improve the production of 2‐phenylethanol (commonly used as rose scent) in a two‐species community comprising *E. coli* and *Meyerozyma guilliermondii* (Figure 1G) (Ou et al., 2022). The authors use *E. coli* to convert glucose into 1‐phenylalanine, which is then further transformed into 2‐phenylethanol by *M. guilliermondii*. When the two species were separately encapsulated in connected micron‐sized hydrogel particles, a 6‐fold increase was seen in 2‐phenylethanol production, as compared to when these species were co‐encapsulated within the same connected droplets (Figure 1H). Both betaxanthin and 2‐phenylethanol are costly to extract from plants and more sustainable synthesis routes are much sought after (Guerrero‐Rubio et al., 2019; Wang et al., 2019). These two examples not only show that microbial communities can synergistically produce these chemicals as an alternative to plant extraction, but that one can optimise these processes by manipulating the spatial structure of the microbial communities with 3D printing.

Looking forward, 3D printers show great promise as a tool for understanding the ecological rules that govern microbial communities. There is a large body of theory—much of it first developed with plants in mind (Tilman et al., 2014)—that seeks to understand fundamental questions in community ecology, such as what makes a productive (high number of cells) and stable (resilient to perturbation) community? Such questions are equally important for our understanding of microbial communities. For example, it is increasingly clear that a diverse (high number of species), and stable gut microbiota is correlated with disease prevention (Faith et al., 2013; Lozupone et al., 2012). However, we do not yet understand what generates diversity and stability in such systems. There are some clear predictions: (1) diverse communities should help prevent pathogen invasion by ensuring most of the available nutrients are utilised and thus unavilable for pathogens (Bell et al., 2005), and (2) coarse spatial structures can promote stability by limiting the strength of competition between community members (Coyte et al., 2015; Krishna Kumar et al., 2021; Nadell et al., 2016). However, unlike plant communities, the spatial scale at which diversity and structure need to be studied in microbes is often tiny, which is why printing technologies can be so valuable. If such fundamental questions can be addressed, one can in principle apply these findings to optimise any kind of microbial community. Indeed, many of the most important microbiome communities involve interactions with the cells of a eukaryotic host, such as occurs in the human microbiome. An important goal therefore is to be able to print both microbes and eukaryotic cells in combination, to generate more realistic microbiome models.

The application of 3D printing to microbial communities offers exciting opportunities across healthcare, agricultural, and industrial settings. By bringing the scale at which we work down to the scale at which microbes actually operate, there is hope to truly understand these tiny ecological systems that influence so many aspects of our lives.

## AUTHOR CONTRIBUTIONS

**R.K.K.** and **K.R.F.** conceived, wrote, and edited the opinion piece.

## FUNDING INFORMATION

R.K.K. was funded by the Health Research Bridging Salary Scheme (0011044) at the University of Oxford. K.R.F. is funded by a European Research Council Advanced Grant (787932) and a Wellcome Trust Investigator Award (209397/Z/17/Z).

## CONFLICT OF INTEREST

K.R.F. is a co‐founder of Postbiotics plus research LLC.
